# Complete Chloroplast Genomes Provide Insights Into Evolution and Phylogeny of *Campylotropis* (Fabaceae)

**DOI:** 10.3389/fpls.2022.895543

**Published:** 2022-05-18

**Authors:** Yu Feng, Xin-Fen Gao, Jun-Yi Zhang, Li-Sha Jiang, Xiong Li, Heng-Ning Deng, Min Liao, Bo Xu

**Affiliations:** ^1^CAS Key Laboratory of Mountain Ecological Restoration and Bioresource Utilization and Ecological Restoration and Biodiversity Conservation Key Laboratory of Sichuan Province, Chengdu Institute of Biology, Chinese Academy of Sciences, Chengdu, China; ^2^College of Life Sciences, University of Chinese Academy of Sciences, Beijing, China; ^3^Mangkang Ecological Station, Tibet Ecological Safety Monitor Network, Changdu, China

**Keywords:** *Campylotropis*, legume, adaptive evolution, phylogenomics, comparative genomics, chloroplast genome

## Abstract

The genus *Campylotropis* Bunge (Desmodieae, Papilionoideae) comprises about 37 species distributed in temperate and tropical Asia. Despite the great potential in soil conservation, horticulture, and medicine usage, little is known about the evolutionary history and phylogenetic relationships of *Campylotropis* due to insufficient genetic resources. Here, we sequenced and assembled 21 complete chloroplast genomes of *Campylotropis* species. In combination with the previously published chloroplast genomes of *C. macrocarpa* and closely related species, we conducted comparative genomics and phylogenomic analysis on these data. Comparative analysis of the genome size, structure, expansion and contraction of inverted repeat (IR) boundaries, number of genes, GC content, and pattern of simple sequence repeats (SSRs) revealed high similarities among the *Campylotropis* chloroplast genomes. The activities of long sequence repeats contributed to the variation in genome size and gene content in *Campylotropis* chloroplast genomes. The *Campylotropis* chloroplast genomes showed moderate sequence variation, and 13 highly variable regions were identified for species identification and further phylogenetic studies. We also reported one more case of *matK* pseudogene in the legume family. The phylogenetic analysis confirmed the monophyly of *Campylotropis* and the sister relationship between *Lespedeza* and *Kummerowia*, the latter two genera were then sister to *Campylotropis*. The intrageneric relationships of *Campylotropis* based on genomic scale data were firstly reported in this study. The two positively selected genes (*atpF* and *rps19*) and eight fast-evolving genes identified in this study may help us to understand the adaptation of *Campylotropis* species. Overall, this study enhances our understanding of the chloroplast genome evolution and phylogenetic relationships of *Campylotropis*.

## Introduction

The genus *Campylotropis* Bunge belongs to the tribe Desmodieae (Benth.) Hutchinson in the legume subfamily Papilionoideae. It comprises *c*. 37 species of deciduous shrubs and subshrubs that distributed in Asia from the Himalaya region through Southeast Asia to China and Korea (Barham, 1997; Iokawa and Ohashi, 2008; Huang et al., 2010). Southwest China is the diversity center of *Campylotropis* as it harbors *c*. 80% of the species, and *c*. 20 species are endemic to this region (Iokawa and Ohashi, 2008). Most species in this genus have important value in soil conservation due to their tolerance of arid soils (Huang et al., 2010). Some *Campylotropis* species are also valuable for horticulture and medicine usage. For example, *C. polyantha* is widely used in gardening due to its numerous racemes of showy flowers and long-lasting fluorescence (Barham, 1997). The dried roots of *C. hirtella* can be used as traditional Chinese medicine for the treatment of benign prostate hyperplasia (Wen et al., 2007), and *C. trigonoclada* contains daucosterol linoleate which can be used for the treatment of breast cancer (Han et al., 2018).

As suggested by previous molecular phylogenetic studies, *Campylotropis* is sister to the other two genera of subtribe Lespedezinae (i.e., *Lespedeza* and *Kummerowia*) in tribe Desmodieae (Xu et al., 2012; Jabbour et al., 2018; Jin et al., 2019). Much effort has been made to clarify species relationships within *Campylotropis*, mostly based on morphological characters such as leaf and calyx morphology (e.g., Iokawa and Ohashi, 2008; Huang et al., 2010). However, most of the morphological characteristics (e.g., persistence of bracts, the color of flowers, and shape of keel petals) are polymorphic and vary continuously among species, causing controversial species delimitation in this genus (Iokawa and Ohashi, 2008). Besides, little is known about its intrageneric and interspecific relationships due to the lack of comprehensive molecular phylogenetic studies.

Chloroplasts, derived from photosynthetic bacteria, play critical roles in the survival, adaptation, and evolution of plants (Wicke et al., 2011; Zhao et al., 2019; Dopp et al., 2021). Although the chloroplast (cp) genomes are much smaller than most nuclear genomes, they encode essential proteins related to photosynthesis, fixation of carbon and nitrogen, and biosynthesis of starch, pigments, fatty acids, and amino acids (Howe et al., 2003; Wicke et al., 2011; Daniell et al., 2016). Chloroplast genomes have relatively stable structure and gene content compared to nuclear genomes. The typical structure of angiosperm cp genome is a circular double-stranded DNA molecule, exhibiting a conserved quadripartite structure [i.e., two inverted repeats (IRs) separated by a large single-copy region (LSC) and a small single-copy region (SSC)] and containing 110–130 genes (Sugiura, 1992; Daniell et al., 2016). The characteristics of cp genomes including lack of recombination, low nucleotide substitution rates, and usually uniparental inheritance make them the primary source to explore phylogenetic evolution of plant species (Shaw et al., 2005). Besides, structural variants such as expansion and contraction of IRs, gains or losses of genes and introns, and dynamics of repeat sequences (e.g., simple sequence repeat, SSR) provide resources for evaluating genomic evolutionary history (e.g. Sabir et al., 2014; Keller et al., 2017). The development of sequencing technology and analysis tools makes the acquisition of cp genomes much easier than before, thus promptly extending gene-based phylogenetics to phylogenomics (Lu et al., 2017). In fact, recent phylogenomic studies have been successful in reconstructing phylogenies at various taxonomic scales (e.g., genera and families) across angiosperms using the cp genome datasets (e.g., Cai et al., 2015; Ruhsam et al., 2015; Luo et al., 2016; Zhang et al., 2017, 2021).

Here, we present 21 complete cp genomes of *Campylotropis* species assembled from Illumina short reads. In combination with the previously published cp genomes of *C. macrocarpa* (Jin et al., 2019) and closely related species, we conducted comparative genomics and phylogenomic analyses on these data with the following aims: (1) to reveal the global structural patterns of *Campylotropis* cp genomes; (2) to investigate variations of SSRs and repeat sequences among *Campylotropis* cp genomes; (3) to screen highly variable regions suitable for species identification and phylogenetic studies; (4) to reconstruct a robust phylogenetic relationship within *Campylotropis* and among genera in the tribe Desmodieae; and (5) to investigate adaptive evolution patterns of cp genes in *Campylotropis*. These results will provide insights into the evolutionary history of *Campylotropis* and tribe Desmodieae as well as abundant information for future phylogenetic and population genetic studies.

## Materials and Methods

### Taxon Sampling, DNA Extraction, and Sequencing

In this study, leaf materials of 21 accessions representing 17 *Campylotropis* species (including four subspecies, one variety, and one forma) were collected from the field and preserved in silica gel (Table 1). Voucher specimens were deposited in the Herbarium of the Chengdu Institute of Biology (CDBI; Supplementary Table S1). The extraction of total genomic DNA, library preparation, and Illumina sequencing for each accession were described in our previous study (Liao et al., 2021).

**Table 1:** Characteristics of the 22 complete chloroplast genomes for *Campylotropis*, including 21 newly generated accessions and the previously published accession of *Campylotropis macrocarpa*.

Sample code	Species name	Size (bp)				GC content (%) total (LSC/SSC/IR)	No. of genes (PCGs/tRNA/rRNA)	GenBank accession	Sample location
		Total	Large single-copy region (LSC)	Small single-copy region (SSC)	Inverted repeat (IR)				
xubo1489	*Campylotropis albopubescens*	149,165	82,871	18,854	23,720	34.84 (32.23/27.94/42.15)	128 (83/37/8)	OM775444	China. Yunnan: Shiping
S867	*Campylotropis bonii*	153,122	82,869	18,899	25,677	34.98 (32.31/28.02/41.84)	129 (82/39/8)	OM775455	China. Guangxi: Jingxi
XB-DR-C	*Campylotropis brevifolia*	148,855	82,648	18,805	23,701	34.83 (32.23/27.90/42.13)	128 (83/37/8)	OM775434	China. Yunnan: Derong
xubo1390	*Campylotropis capillipes*	152,978	82,903	18,701	25,687	34.95 (32.24/28.13/41.81)	130 (83/39/8)	OM775435	China. Yunnan: Binchuan
xubo1445	*Campylotropis delavayi*	149,088	82,797	18,851	23,720	34.87 (32.28/27.92/42.15)	128 (83/37/8)	OM775436	China. Yunnan: Heqing
xubo1424	*Campylotropis grandifolia*	149,165	82,871	18,854	23,720	34.84 (32.23/27.94/42.15)	128 (83/37/8)	OM775437	China. Yunnan: Mile
xubo1429	*Campylotropis harmsii*	149,291	82,992	18,859	23,720	34.86 (32.23/28.01/42.16)	128 (83/37/8)	OM775438	China. Yunnan: Jinhong
xubo1483	*Campylotropis henryi*	149,153	82,851	18,904	23,699	34.89 (32.30/28.01/42.17)	128 (83/37/8)	OM775440	China. Yunnan: Xinping
xubo1375	*Campylotropis howellii*	149,312	82,965	18,823	23,762	34.81 (32.17/27.92/42.13)	128 (83/37/8)	OM775439	China. Yunnan: Tengchong
xubo1430	*Campylotropis latifolia*	149,176	82,881	18,855	23,720	34.84 (32.23/27.93/42.15)	128 (83/37/8)	OM775441	China. Yunnan: Shiping
--	*Campylotropis macrocarpa*	148,814	82,566	18,808	23,720	34.86 (32.27/27.89/42.14)	128 (83/37/8)	NC_044100	Jin et al., 2019
xubo1425	*Campylotropis cytisoides* f. *parviflora*	148,932	82,655	18,846	23,715	34.83 (32.19/27.93/42.15)	128 (83/37/8)	OM775442	China. Yunnan: Jinhong
xubo1426	*Campylotropis pinetorum* subsp. *velutina*	149,227	82,933	18,848	23,723	34.86 (32.24/28.02/42.16)	128 (83/37/8)	OM775443	China. Yunnan: Eshan
xubo1447	*Campylotropis polyantha*	149,191	82,810	18,941	23,720	34.84 (32.25/27.84/42.16)	128 (83/37/8)	OM775447	China. Yunnan: Dali
xubo1427	*Campylotropis polyantha* var. *tomentosa*	149,001	82,772	18,801	23,714	34.83 (32.22/27.88/42.15)	128 (83/37/8)	OM775445	China. Sichuan: Shimian
xubo1481	*Campylotropis capillipes* subsp*. prainii*	149,092	82,892	18,746	23,727	34.88 (32.25/28.09/42.16)	128 (83/37/8)	OM775446	China. Yunnan: Eshan
xubo1406	*Campylotropis teretiracemosa*	149,169	82,868	18,863	23,719	34.82 (32.17/28.04/42.16)	128 (83/37/8)	OM775449	China. Sichuan: Yanyuan
xubo1428	*Campylotropis thomsonii*	148,963	82,676	18,822	23,732	34.85 (32.23/27.94/42.15)	128 (83/37/8)	OM775450	China. Yunnan: Mengla
xubo1393	*Campylotropis trigonoclada*	149,227	82,957	18,840	23,715	34.83 (32.18/28.05/42.17)	128 (83/37/8)	OM775451	China. Yunnan: Binchuan
xubo1407	*Campylotropis wilsonii*	149,113	82,771	18,870	23,736	34.85 (32.26/27.90/42.13)	128 (83/37/8)	OM775452	China. Sichuan: Wenchuan
xubo1434	*Campylotropis yunnanensis* subsp*. filipes*	149,122	82,822	18,862	23,719	34.84 (32.24/27.90/42.14)	128 (83/37/8)	OM775453	China. Sichuan: Panzhihua
xubo1435	*Campylotropis yunnanensis*	148,548	82,269	18,841	23,719	34.90 (32.32/27.95/42.14)	128 (83/37/8)	OM775454	China. Yunnan: Yongsheng

### Chloroplast Genome Assembly, Annotation, and Comparison

For each accession, ~25 Gb of raw data were generated with pair-end 150 bp read length. Trimmomatic v0.39 (Bolger et al., 2014) was used to remove low-quality and adapter-containing reads. The clean data were then assembled using GetOrganelle v1.7.5 (Jin et al., 2020). Plastid Genome Annotator (Qu et al., 2019) was used to annotate the cp genomes based on one published accession of *Campylotropis* (*C. macrocarpa*; NC_044100; Jin et al., 2019) and 15 accessions of closely related legume species (Supplementary Table S2). Manual corrections for start and stop codons and the determination of pseudogenes were performed in Geneious v11 (Biomatters Ltd., Auckland, New Zealand). For the *matK* pseudogene annotated in the cp genome of *C. bonii* (see section “Results”), we further mapped raw reads to the assembled sequence of the *matK* gene, and performed Sanger sequencing to validate the accuracy of the assembled sequence. Raw reads were remapped to 400-bp surroundings of the IRb ends to quantify the IR junctions. Genome map of the cp genomes was generated using the online OrganellarGenome DRAW tool (OGDRAW; Lohse et al., 2013). To compare the contraction and expansion of IRs among cp genomes of *Campylotropis* and closely related genera, we identified and visualized boundaries of LSC, SSC, and IRs of the 25 whole cp genomes (including 22 *Campylotropis* accessions, two *Lespedeza* accessions, and *Kummerowia striata*) using IRscope (Amiryousefi et al., 2018).

### Repeat Sequence Analysis

For 21 newly generated cp genomes and the published accession of *C. macrocarpa*, SSRs were identified using MISA software (Beier et al., 2017) with parameter settings of 11 for mono-, 6 for di-, 5 for tri-, 4 for tetra-, and 3 for penta- and hexa-nucleotide SSRs. For each of the 22 *Campylotropis* cp genomes, forward, reverse, palindrome, and complementary repeat sequences in LSC, IRb, and SSC regions were identified using REPuter program (Kurtz et al., 2001).

### Molecular Marker Identification

The 22 whole cp genomes were firstly aligned using MAFFT v7 (Katoh and Standley, 2013). To identify hypervariable regions that can be used in species identification and phylogenetic studies for *Campylotropis*, nucleotide diversity (Pi) values were calculated in sliding windows along the alignment with a window length of 600 bp and step size of 200 bp. Pi values of each window were calculated using a custom Python script,^1^ with the formula referring to the algorithm implemented in pixy (Korunes and Samuk, 2021) to obtain unbiased estimations of nucleotide diversity in the presence of alignment gaps. Adjacent windows with a Pi value > 0.01 and a number of parsimony informative sites >25 were joined together as one single hypervariable region. The number of singleton variable sites, number of parsimony informative sites, and Pi values were calculated for each hypervariable region using the custom Python script.

### Phylogenetic Analysis

To estimate the cp-genome-based phylogenetic relationships of *Campylotropis* as well as the tribe Desmodieae, we included the whole cp genomes of 22 *Campylotropis* accessions and 15 outgroups (Supplementary Table S2). The phylogenetic analyses were performed using Maximum likelihoods (ML) and Bayesian inference (BI) methods based on both whole cp genomes and shared protein-coding genes (PCGs). For the former dataset, MAFFT v7 was used to obtain the alignment of 37 whole cp genomes. As for the latter dataset, the shared PCGs were extracted and translated into amino acid sequences, and ClustalW2 (Larkin et al., 2007) was used to align the amino acid sequences. The codon alignment of each PCGs was obtained using PAL2NAL (Suyama et al., 2006). The ML trees were inferred using RAxML v8 (Stamatakis, 2014) based on the alignment of 37 whole cp genomes and the concatenated matrix of 72 PCGs. For each RAxML analysis, GTRGAMMA + I was set as the nucleotide substitution model and 1,000 bootstrap replicates were conducted to determine branch support. The BI analyses were performed using MrBayes v3.2 (Ronquist et al., 2012) with the nucleotide substitution model GTR + G + I (lset nst = 6 rates = invgamma). For each analysis, the posterior probability was estimated with two independent Markov Chain Monte Carlo (MCMC) chains (10 million generations and sampled every 1,000 generations) with the preliminary 25% of sampled data discarded as burn-in.

### Analysis of Selective Pressure

To explore the selective pressure of PCGs in *Campylotropis*, the CODEML program implemented in the PAML v4.9 package (Yang, 2007) was used to estimate the rate of non-synonymous (*d*_N_) and synonymous (*d*_S_) substitutions for PCGs. In general, the ratio of *d*_N_/*d*_S_ (ω) was supposed to equal 1 when under neutral evolution, a larger ω indicates higher positive selection pressure, while a smaller ratio of ω indicates higher pressure of negative selection.

All the 37 accessions in the above phylogenetic analysis were included, and the resulting phylogenetic tree was used as the input topology for CODEML. The codon-wise alignments of nucleotide sequences, which were used as the input sequences for CODEML, were generated with PAL2NAL (Suyama et al., 2006) guided by the peptide alignments. To determine whether each shared PCG has undergone a different evolutionary force in different lineages, we ran branch-site models with a one-ratio model (null hypothesis; ω_0_) in which all branches share the same ω and a two-ratio model in which the foreground branches (*Campylotropis* spp.; ω_f_) have a different ω (alternative hypothesis; ω_b_). Likelihood ratio tests with *χ*^2^ distribution were used to determine whether the alternative hypothesis significantly differ from the null hypothesis (Chi-square test, *p* < 0.05).

## Results

### Characteristics of *Campylotropis* cp Genomes

In this study, a total of 21 whole cp genomes of *Campylotropis* were newly generated and were submitted to GenBank under the accession numbers list in Table 1. Taken together with the previously published one of *C. macrocarpa* (NC_044100), the whole cp genomes of *Campylotropis* ranged from 148,548 bp (*C. yunnanensis*) to 153,122 bp (*C. bonii*), exhibiting a typical quadripartite structure comprising two IR regions (IRa and IRb) of 23,699–25,687 bp, an LSC region of 82,269–82,992 bp, and an SSC region of 18,746–18,941 bp (Table 1). The GC contents of the *Campylotropis* cp genomes were similar (34.81%–34.93%; Table 1). The IRs have the highest GC content (41.81%–42.18%), followed by the LSC region (32.17%–32.32%), and the SSC region (27.84%–28.13%).

**Figure 1:**
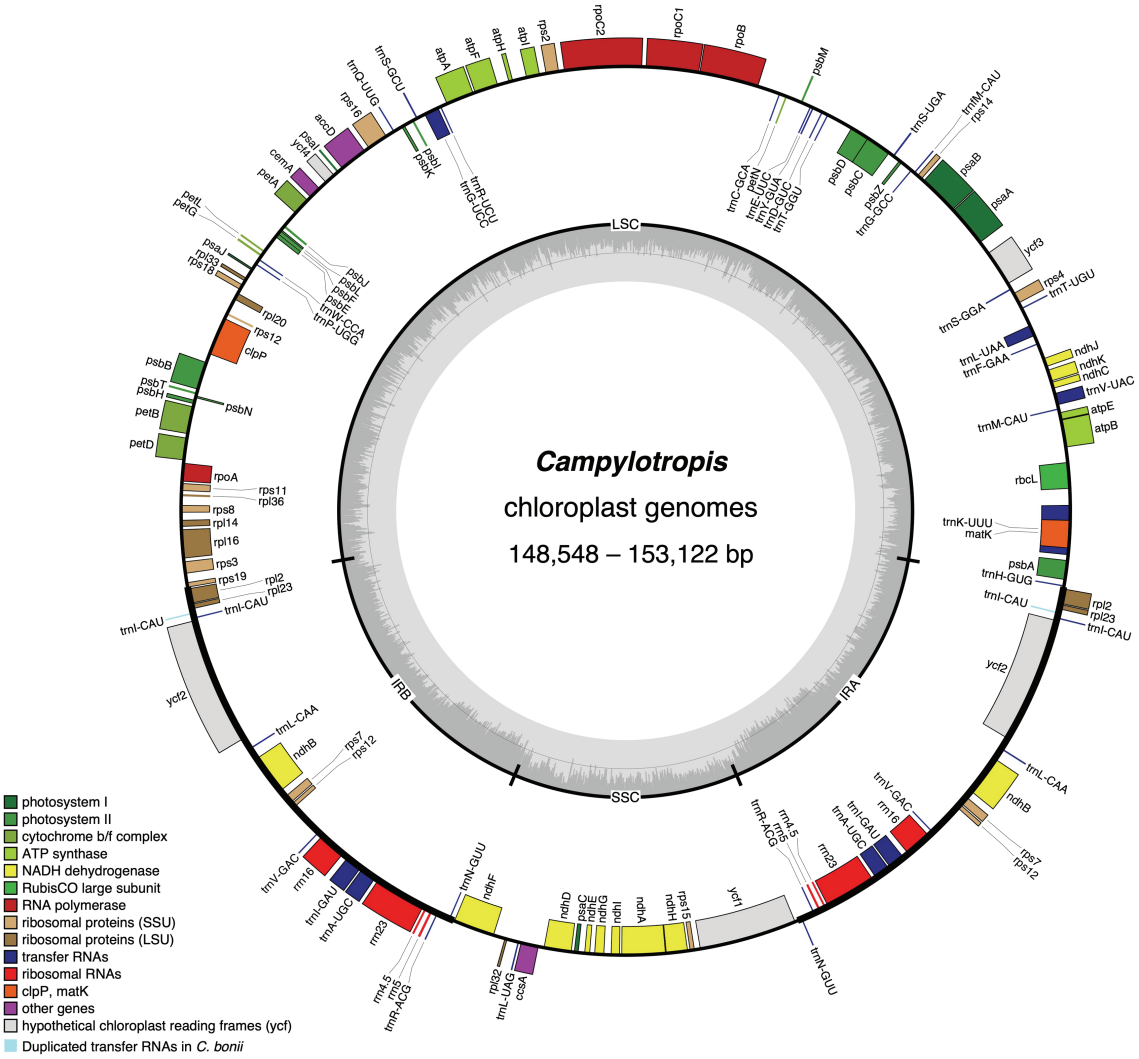
The chloroplast genome map of *Campylotropis* species. Genes inside and outside of the circle are transcribed clockwise and counterclockwise, respectively. Genes belonging to different functional groups are shown in different colors, with extra duplicated genes in *Campylotropis bonii* highlighted in light blue. The dark gray area in the inner circle denotes GC content while the light gray corresponds to the AT content of the genome. LSC, large single copy; SSC, small single copy; and IR, inverted repeat.

The *Campylotropis* cp genomes were similar in gene contents, most of which encode 128 genes, including 83 PCGs, 37 tRNA genes, and eight rRNA genes (all located in the IRs; Table 1; Figure 1). Three species had a few pseudogenes and/or duplicated genes (Table 2). Specifically, *C. capillipes* and *C. bonii* has two more copies of the *trnI-CAU* gene, and *C. bonii* has a pseudogene (*ψmatK*; Table 2), which was confirmed by both raw reads mapping and Sanger sequencing (see Supplementary Figure S1 and Supplementary Dataset). Among the 83 PCGs, 77 were unique, and six (*ndhB*, *rpl12*, *rpl23*, *rps7*, *rps12*, and *ycf2*) were duplicated due to their location in the IRs. Likewise, 30 of the tRNA genes are unique, while seven tRNA genes (*trnA-UGC, trnI-CAU, trnI-GAU, trnL-CAA, trnN-GUU, trnR-ACG*, and *trnV-GAC*) and all four rRNA genes (*rrn23*, *rrn16*, *rrn5*, and *rrn4.5*) were duplicated. Eight PCGs (*petB*, *petD*, *atpF*, *ndhB*, *ndhA*, *rpoC1*, *rpl16*, and *rps16*) and six tRNA genes (*trnA-UGC*, *trnI-GAU*, *trnG-UCC*, *trnL-UAA*, *trnV-UAC*, *trnK-UUU*) contained one intron, while only three PCGs (*rps12*, *ycf3*, and *clpP*) contained two introns (Table 2). In all newly generated *Campylotropis* cp genomes, the 5′ end of the *rps12* gene was located in the LSC region, and the 3′ end was duplicated in the IRs.

**Table 2:** Summary of gene contents present in the *Campylotropis* chloroplast genomes.

Group of genes	Name of genes
Ribosomal RNAs	*rrn16*(x 2), *rrn23*(x 2), *rrn4.5*(x 2), *rrn5*(x 2) *trnA-UGC* (1)(x 2), *trnI-GAU* (1)(x 2), *trnL-CAA*(x 2), *trnN-GUU*(x 2), *trnR-ACG* (x 2), *trnV-GAC* (x 2), *trnI-CAU* (x 2)^*^, *trnL-UAG*, *trnP-UGG*, *trnW-CCA*, *trnQ-UUG*, *trnS-GCU*, *trnG-UCC* (1), *trnR-UCU*, *trnC-GCA*, *trnE-UUC*, *trnY-GUA*, *trnD-GUC*, *trnT-GGU*, *trnS-UGA*, *trnG-GCC*, *trnfM-CAU*, *trnS-GGA*, *trnT-UGU*, *trnL-UAA* (1), *trnF-GAA*, *trnV-UAC* (1), *trnM-CAU*, *trnK-UUU*(1), *trnH-GUG*
Transfer RNAs	
Proteins of small ribosomal subunit	*rps2*, *rps3*, *rps4*, *rps7* (x 2), *rps8*, *rps12* (2)(x 2), *rps14*, *rps15*, *rps18*, *rps19*, *rps16* (1)
Proteins of large ribosomal subunit	*rpl2* (x 2), *rpl14*, *rpl16*(1), *rpl20*, *rpl23*(x 2), *rpl32*, *rpl33*, *rpl36*
Subunits of RNA polymerase	*rpoA*, *rpoB*, *rpoC1* (1), *rpoC2*
Subunits of photosystem I	*psaA*, *psaB*, *psaC*, *psaI, psaJ*
Subunits of photosystem II	*psbA*, *psbB*, *psbC*, *psbD*, *psbE*, *psbF*, *psbH*, *psbI*, *psbJ*, *psbK*, *psbL*, *psbM*, *psbN*, *psbT*, *psbZ*
Subunits of ATP synthase	*atpA*, *atpB*, *atpE*, *atpF*(1), *atpH*, *atpI*
Subunits of cytochrome b/f complex	*petA*, *petB*(1), *petD*(1), *petG*, *petL*, *petN*
Subunits of NADH-dehydrogenase	*ndhA* (1), *ndhB*(1)(x 2), *ndhC*, *ndhD*, *ndhE*, *ndhF*, *ndhG*, *ndhH*, *ndhI*, *ndhJ*, *ndhK*
Large subunit of RuBisco	*rbcL*
Acetyl-CoA carboxylase	*accD*
Cytochrome c biogenesis	*ccsA*
Envelope membrane protein	*cemA*
Maturase	*matK* ^**^
Protease	*clpP*(2)
Conserved hypothetical chloroplast reading frames	*ycf1*, *ycf2* (x 2), *ycf3*(2), *ycf4*

*(1) Genes with one intron; (2) Genes with two introns; (x 2) Genes with two copies*.

* **Campylotropis bonii* and *Campylotropis capillipes* have four copies of trnI-CAU*.

** *The matK gene is a pseudogene in *Campylotropis bonii**.

### Comparative Analysis of IR Boundaries

The IR boundary of the assembled cp genomes were quantified by the remapping of short reads, which showed above 300× for the IRb ends and surrounding areas (Supplementary Table S3). We compared the IR boundaries of 25 cp genomes from subtribe Lespedezinae, including *Lespedeza maritima*, *Lespedeza cuneata*, *Kummerowia striata*, and 22 *Campylotropis* accessions, and found a little variation of the expansion/contraction of the IRs (Supplementary Figure S2). The JLA (IRa-LSC) and JSA (IRa-SSC) boundaries are highly consistent in the 25 cp genomes, with the former located between *rpl2* and *trnH*, and the latter between *ycf1* and *trnN*. The distances between the JLA boundary and *trnH* were 0–19 bp, while those between the JSA boundary and *ycf1* varied from 128 to 144 bp. The JLB (IRb-LSC) boundaries cut through *rps19* in most species, with 32–48 bp of *rps19* extended into the IRb, while the JLB boundaries of *C. thomsonii* and *C. parviflora* were 88 bp away from *rps19* due to the contraction of IRs. The distance between the JSB (IRb-SSC) boundaries and *ndhF* varied from 2 to 33 bp in most species except *Kummerowia striata*, where *ndhF* extended 11 bp into IRb due to the expansion of IRs.

### Characteristics of Repeat Sequences

**Figure 2:**
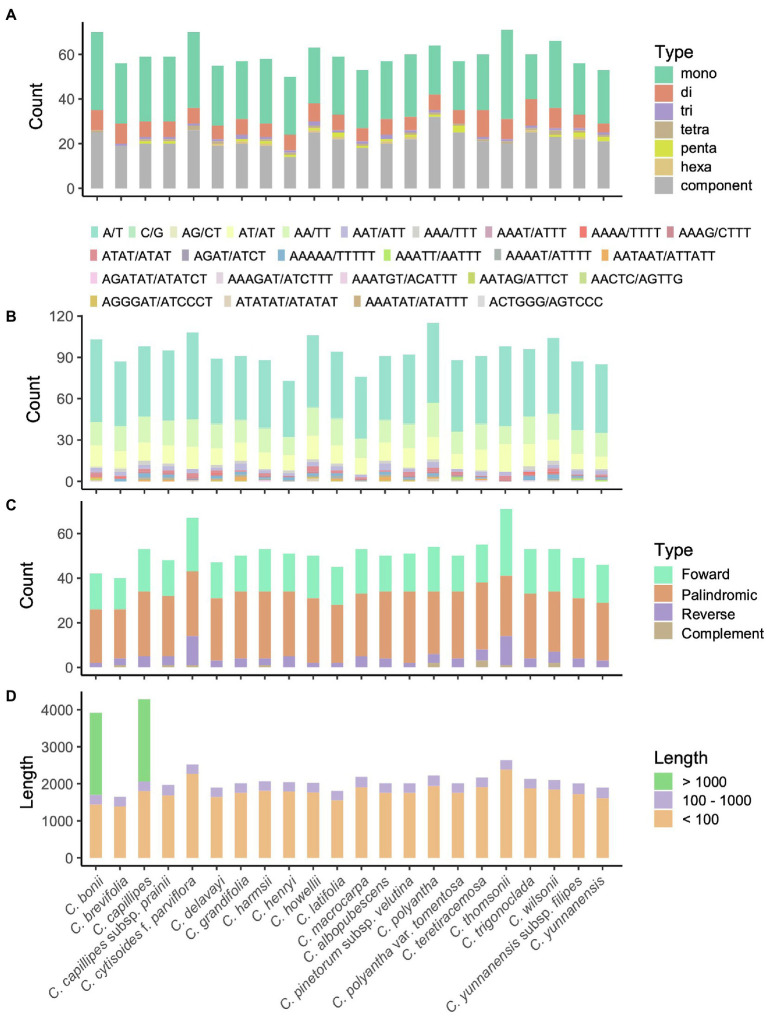
Patterns of simple sequence repeats (SSRs; **A,B**) and long sequence repeats (LSRs; **C,D**) for the 23 chloroplast genomes of *Campylotropis* species. **(A)** Number of motifs and their abundance of SSRs in each species. **(B)** Type of motifs and their abundance of SSRs in each species. **(C)** Type and abundance of LSRs in each species. **(D)** Accumulative length of LSRs in each species.

The number of SSRs in the *Campylotropis* species varied from 50 in *C. harmsii* to 115 in *C. teretiracemosa* (Supplementary Table S4), in which mononucleotide SSRs were most abundant, followed by component and dinucleotides SSRs (Figure 2A). Among the motifs in the SSRs, A/T, AA/TT, and AT/AT were the most frequently occurring motifs (Figure 2B). Besides, most of the SSRs were located in the LSC (38–56) and SSC (10–18) regions, and very few were located in the IRs (Supplementary Table S5). REPuter identified 40–71 repeat sequences with length > 30 bp, covering 1,647–4,278 bp in the cp genomes of *Campylotropis* species (Figures 2C,D). Palindromic repeat sequences were most abundant (22–32), followed by forward (14–20) and reverse (2–13) repeat sequences (Figure 2C; Supplementary Table S6). All the repeat sequences with length > 30 bp were located in LSC (33–64) and IRs (6–10), while none of them were identified in the SSC region (Supplementary Table S6). Most of the repeat sequences were less than 100 bp, a few of them were larger than 100 bp (Figure 2D; Supplementary Table S6). Notably, *C. bonii* and *C. capillipes* each had a forward repeat sequence with a length of 2,219 and 2,217 bp, respectively (Figure 2D; Supplementary Table S7). Both repeat sequences were located between *rpl23* and *ycf2* in the IRs, which caused the duplication of *trnI-CAU* and resulted in four copies of this gene (Supplementary Figure S3).

### Identification of Candidate Molecular Markers

**Figure 3:**
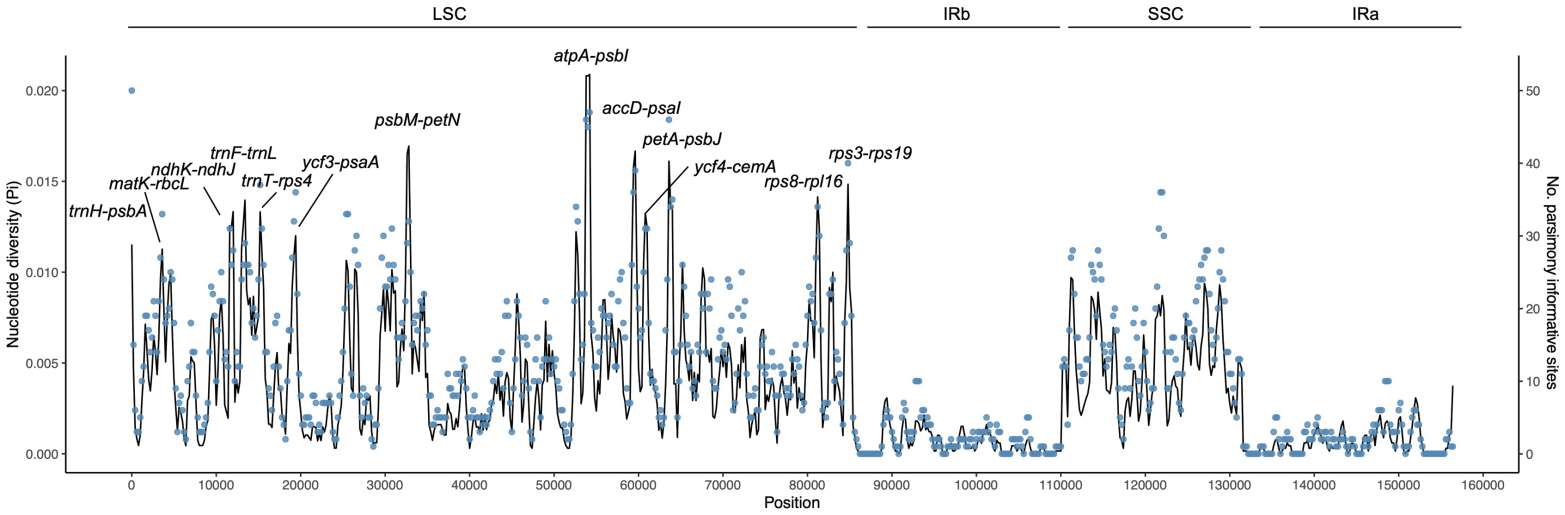
Nucleotide diversity (Pi, black line, vertical left axis) and number of parsimony informative sites (blue dots, vertical right axis) of the *Campylotropis* chloroplast genomes based on sliding window analysis. The window length is 600 bp and the step size is 200 bp. The horizontal axis indicates the position of the midpoint of a window. The 13 regions with high diversity are indicated above the peaks.

Using sliding window analysis, we found that most genetic variations in the cp genomes of *Campylotropis* occurred in the LSC and SSC regions (Figure 3). A total of 13 intergenic spacer regions located in the LSC region, ranging from 547 to 1,995 bp, were identified as potential molecular markers for phylogenetic and population genetic studies (Figure 3; Table 3). Among them, the intergenic spacer of *atpA* and *psbI* (*atpA*-*psbI*) was the longest (1,995) and contained the greatest number of parsimony informative sites (109), while the intergenic spacer of *ycf4* and *cemA* (*ycf4*-*cemA*) had the highest Pi value (0.0117).

**Table 3:** Hypervariable regions identified among the 22 cp genomes of *Campylotropis*.

Start	End	Length	# SVS	# PIP	Pi	Gene name
0	547	547	101	50	0.01154744	*trnH-psbA*
3,248	4,977	1,729	141	72	0.00815403	*matK-rbcL*
11,237	12,215	978	61	39	0.00847844	*ndhK-ndhJ*
12,770	13,801	1,031	241	58	0.01136576	*trnF-trnL*
14,560	15,451	891	158	49	0.01037634	*trnT-rps4*
18,421	19,380	959	71	43	0.0078191	*ycf3-psaA*
31,452	32,596	1,144	116	44	0.01010241	*psbM-petN*
51,126	53,121	1,995	269	109	0.01122222	*atpA-psbI*
57,121	58,064	943	84	46	0.01088579	*accD-psaI*
58,636	59,422	786	48	37	0.01167507	*ycf4-cemA*
61,206	62,172	966	77	52	0.01052879	*petA-psbJ*
78,264	79,190	926	147	42	0.00917555	*rps8-rpl16*
81,692	82,566	874	163	55	0.01090692	*rps3-rps19*

*The start and end positions are referred to *Campylotropis macrocarpa*. # SVS: number of singleton variable sites; # PIP: number of parsimony informative sites*.

### Phylogenetic Relationships of *Campylotropis*

**Figure 4:**
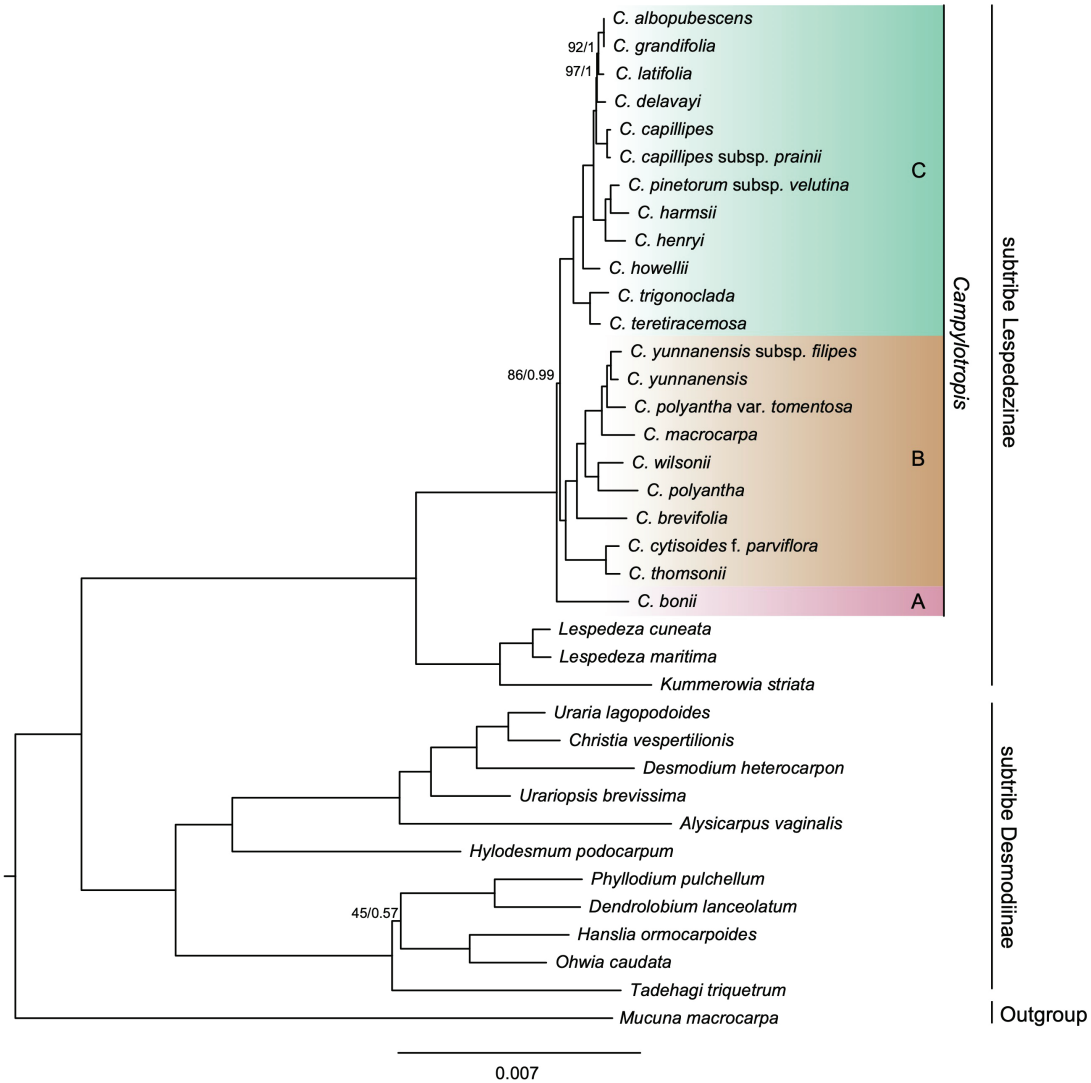
Phylogenetic tree obtained using the Maximum Likelihood (ML) and Bayesian Inference (BI) method for *Campylotropis* spp. and closely related species based on whole cp genomes. Numbers above branches indicate ML bootstrap supports (BS; before the slash) and Bayesian posterior probabilities (PP; after the slash). The full support values are not indicated.

The phylogenetic trees inferred from Maximum likelihood (ML) and Bayesian inference (BI) based on the whole cp genome shared an identical topology and showed little differences in support values (Figure 4). The concatenated alignment of PCGs resulted in similar topologies, with a few differences with regard to the relationships within *Campylotropis* (Supplementary Figures S4, S5). All topologies fully supported the reciprocal monophyly of the two subtribes in tribe Desmodieae [100% bootstrap support (BS) and 1 posterior probability (PP)]. In the subtribe Lespedezinae, *Kummerowia striata* and the two *Lespedeza* species formed a clade (BS = 100%, PP = 1), and *Campylotropis* was also a monophyletic clade (BS = 100%, PP = 1).

As for the relationship within *Campylotropis*, both ML and BI trees based on the whole cp genome supported *C. bonii* (lineage A) as sister to the remaining species (Figure 4), and the latter clade (BS = 86%, PP = 0.99) segregated into two subclades (lineages B and C), each with full support values (BS = 100%, PP = 1). Lineage B included *C. yunnanensis* subsp*. filipes*, *C. yunnanensis*, *C. polyantha* var. *tomentosa*, *C. macrocarpa*, *C. wilsonii*, *C. polyantha*, *C. brevifolia*, *C. cytisoides* f*. parviflora*, and *C. thomsonii*. And, lineage C included *C. albopubescens*, *C. grandifolia*, *C. latifolia*, *C. delavayi*, *C. capillipes*, *C. capillipes* subsp*. prainii*, *C. pinetorum* subsp*. velutina*, *C. harmsii*, *C. henryi*, *C. howellii*, *C. trigonoclada*, and *C. teretiracemosa*. The ML three based on the PCGs dataset showed the same topology as that based on the whole cp genome with regard to the relationship among the three subclades of *Campylotropis*, albeit the supporting values were lower (lineage B: BS = 93%; lineage B sister to lineage C: BS = 79%; Supplementary Figure S4). However, the BI inference based on the PCGs dataset revealed a different topology, in which *C. bonii* was weakly supported to be a sister clade of lineage B (PP = 0.604; Supplementary Figure S5).

### Selective Pressure of cp Genes in *Campylotropis*

**Figure 5:**
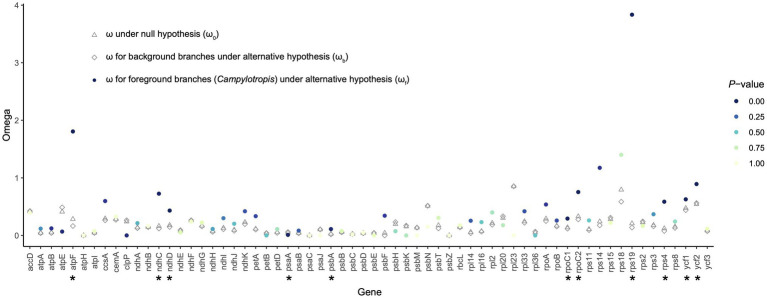
The ratio of non-synonymous (*d*_N_) and synonymous (*d*_S_) substitutions (*d*_N_/*d*_S_; ω) for protein-coding genes shared by the Desmodieae species. The asterisks under the gene names indicate statistical significance (*p* < 0.05) between the null hypothesis and the alternative hypothesis.

A total of 68 shared PCGs were subjected to the selective pressure analysis (Supplementary Table S8). Most of the genes were subjected to purifying selection (ω < 1; Figure 5). Using the likelihood ratio test, we found that 11 genes showed significantly different selective pressure in *Campylotropis* (Figure 5; Supplementary Table S8). Among them, two genes (*atpF* and *rps19*) showed obvious signatures of positive selection (ω_f_ > 1, *p* < 0.05) in *Campylotropis* and eight genes (*ndhC*, *ndhD*, *psbA*, *rpoC1*, *rpoC2*, *rps4*, *ycf1*, and *ycf2*) evolved faster in *Campylotropis* than in the background branches (ω_f_ > ω_b_, *p* < 0.05; Figure 5; Supplementary Table S8).

## Discussion

### Variations and Evolution of Whole cp Genomes in *Campylotropis*

The 21 newly assembled and one previously published *Campylotropis* cp genomes showed little variation in genome structure and genome length, as found in other legume species (Wang et al., 2018; Oyebanji et al., 2020; Zhang et al., 2020; Liao et al., 2021). The *Campylotropis* cp genomes exhibit the typical quadripartite structure and no large structural variant was found (Table 1). The genome length of these species was similar (148,548–153,122 bp) and fell within the range of subfamily Papilionoideae (*c*. 140–160 kb; Oyebanji et al., 2020). Other genome features, including lengths of LSC, SSC, and IRs, expansion and contraction of IR boundaries, number of genes, GC content, the pattern of SSRs also varied little within this genus, which is comparable to other genera from the legume family (e.g., Oyebanji et al., 2020; Liao et al., 2021).

Despite the general homogeneity characteristics mentioned above, there are some interesting inconsistencies worth mentioning in *Campylotropis* cp genomes. Previous studies demonstrated that expansion and contraction of IRs substantially contribute to the change in the size of cp genomes (Ruhlman and Jansen, 2014; Zheng et al., 2017; Gu et al., 2020). In our study, the JLB (IRb-LSC) boundaries cut through *rps19* in most species, except in *C. thomsonii* and *C. parviflora*, where JLB was located between *rps19* and *rpl2*, causing less than 100-bp length variation of the IRs (Supplementary Figure S2). However, the cp genomes of *C. bonii* and *C. capillipes* were 3–4 kb longer than the rest without showing any significant signal of IR expansion (Figure 2; Supplementary Figure S2). Both cp genomes have a ~2 kb long sequence repeat in each IR region, causing a ~4 kb increase in total genome length. These results indicate that similar to nuclear genomes (Bennetzen et al., 2005), dynamics in repeat sequences rather than expansion and contraction of IRs played an important role in the length variation of *Campylotropis* cp genomes. The long sequence repeats also caused duplication of *trnI-CAU* and resulted in four copies of this gene (Supplementary Figure S1; Supplementary Table S7).

The *Campylotropis* cp genomes showed moderate sequence variation, most occurring in the LSC region (Figure 3). Consequently, all 13 candidate molecular markers were located in the LSC region, which may be useful in further studies of species delimitation, phylogenetic, and population genetic studies (Table 3). Many of these molecular markers have been reported in other studies, such as *trnH-psbA* (Li et al., 2021), *accD-psaI* (Chen et al., 2021), and *petN-trnD* (Liao et al., 2021). Notably, the *matK* gene, which encodes a protein essential for *in vivo* splicing of Group II introns (Ahlert et al., 2006), is a pseudogene in *C. bonii*. As one of the most frequently used molecular markers in angiosperm phylogenetic studies (Patwardhan et al., 2014), *matK* has a high overall evolutionary rate in contrast to other chloroplast genes (Wanke et al., 2007). In fact, pseudogenic copies of *matK* pseudogene were reported in orchids (Kocyan et al., 2008), Piperales (Wanke et al., 2007), and Ericaceae (Braukmann et al., 2017). In the legume family, *matK* pseudogenes were found in *Tadehagi triquetrum* (GenBank accession: MW557314.1; unpublished) and reported in *Tylosema* spp. (Wang et al., 2018). Here, *C. bonii* provided one more case for legume plants living with pseudogenic *matK* gene.

### Phylogenetic Relationships

The phylogenetic trees reconstructed on both whole cp genome and shared PCGs in this study fully supported the monophyly of the two subtribes of Desmodieae (Figure 4; Supplementary Figures S4, S5). The subtribe Desmodiinae was divided into two fully supported monophyletic groups as described in previous studies (Jabbour et al., 2018; Jin et al., 2019). Subtribe Lespedezinae consist of three genera: *Campylotropis*, *Lespedeza*, and *Kummerowia* (Figure 4). Since the first Chinese species of *Campylotropis* (*C. macrocarpa*) was described as *Lespedeza macrocarpa* Bunge (Bunge, 1835), a number of species have been recorded under *Lespedeza*, *Campylotropis* was thought to be derived from *Lespedeza* (Fu, 1987). However, molecular phylogenetic studies based on one or several molecular markers found a sister relationship between *Lespedeza* and *Kummerowia* (Xu et al., 2012; Jabbour et al., 2018). Likewise, whole cp genomes in both Jin et al. (2019) and this study confirmed that *Lespedeza* was sister to *Kummerowia*, and the two genera were then sister to *Campylotropis*.

The intrageneric and interspecific relationships of *Campylotropis* have been unsettled for a long time due to complex morphological characteristics and lack of molecular phylogenetic studies (e.g., ﻿ Jabbour et al., 2018). Our results strongly support *Campylotropis* as a monophyletic group, consisting of three lineages (i.e., A, B, and C; Figure 4). Lineage A contains only one species, *C. bonii*, which was sister to all the remaining species of *Campylotropis* (lineage B and lineage C). Species from lineage C were mostly restricted in southwestern China and Southeast Asia, while lineage B contained regional endemic and widely distributed species. For example, among species in lineage B, *C. wilsonii* is endemic to western Sichuan while *C. macrocarpa* is distributed throughout southwestern China and East Asia (Huang et al., 2010). However, the relationships among the three lineages were not resolved, as the support value of the sister relationship between lineage B and lineage C was relatively low (Figure 4; Supplementary Figure S4), and the BI inference resulted in a different topology (Supplementary Figure S5). The former topology agrees with a previous study that included five *Campylotropis* species in the phylogenetic analysis of the tribe Desmodieae, but the results were only based on several molecular markers: ﻿chloroplast (*rbcL*, *psbA-trnH*) and nuclear (ITS-1) DNA sequences (Jabbour et al., 2018). Thus, phylogenetic studies with more extensive sampling and nuclear genomic data are needed to elucidate the intrageneric relationships of *Campylotropis*.

### Selective Pressure

Positive selection is assumed to play key parts in the adaptation of organisms to diverse environments (Moseley et al., 2018), while negative (purifying) selection is a ubiquitous evolutionary force responsible for genomic sequence conservation across long evolutionary timescales (Cvijović et al., 2018). For example, the positive selection pressure of genes related to photosynthesis was found less than other types of genes (Du et al., 2016; Gao et al., 2018; Li et al., 2020). As expected, the ω values for most genes, especially photosynthesis genes, were less than 1, either in *Campylotropis* or in background branches (Figure 5). The two genes under significant positive selection in *Campylotropis*: *atpF* and *rps19* (ω_f_ > 1; *p* < 0.05) were also found under positive selection in other species, e.g., *atpF* in two deciduous *Quercus* species (Yin et al., 2018), and *rps19* in *Garcinia paucinervis* (Wang et al., 2021). As indicated in Yin et al. (2018), *atpF* gene is highly divergent between deciduous and evergreen sclerophyllous oaks since the former loses its leaves in cold and drought seasons. Despite having ω_f_ < 1, eight genes (*ndhC*, *ndhD*, *psbA*, *rpoC1*, *rpoC2*, *rps4*, *ycf1*, and *ycf2*) significantly accelerated their evolution in *Campylotropis* compared to background branches (ω_f_ > ω_b_, *p* < 0.05). Some of them were reported to be under significant positive selection in other taxa, such as *ycf1* in seed plants (Zheng et al., 2017), *ndhC* in *Echinacanthus* (Gao et al., 2019), and *rpoC2* in *Rehmannia* (Zeng et al., 2017). Therefore, these positively selected and fast-evolving genes may play an important role in the adaptation of *Campylotropis* species to arid soils and various types of habitats.

## Conclusion

In this study, we assembled 21 whole cp genomes for *Campylotropis* spp. Comparative analysis of the cp genome size, structure, expansion and contraction of IR boundaries, number of genes, GC content, and pattern of SSRs revealed high similarities among the *Campylotropis* cp genomes. The activities of long sequence repeats contributed to the variation in genome size and gene content in *Campylotropis* cp genomes. The *Campylotropis* cp genomes showed moderate sequence variation, and 13 candidate regions were identified for further studies of species identification and phylogenetic studies. We also reported one more case of *matK* pseudogene for legume species in *C. bonii*. The phylogenetic analysis confirmed the monophyly of *Campylotropis* and the sister relationship between *Lespedeza* and *Kummerowia*, the latter two genera were then sister to *Campylotropis*. And, its intrageneric relationships based on genomic scale data were firstly reported in this study. The two positively selected genes (*atpF* and *rps19*) and eight fast-evolving genes identified in this study may help us to understand the adaptation of *Campylotropis* species.

## Data Availability Statement

The sequences and annotations of the newly generated chloroplast genomes of Campylotropis species were deposited in the National Center for Biotechnology Information (NCBI) GenBank database under the accession numbers list in Table 1.

## Author Contributions

YF, X-FG, and BX conceived and designed the study. BX, H-ND, J-YZ, and ML collected the sample. YF, J-YZ, L-SJ, and XL analyzed the data. YF wrote the manuscript. BX revised the paper. All authors contributed to the article and approved the submitted version.

## Funding

This work was supported by the National Natural Science Foundation of China (grant no. 31570196), the Second Tibetan Plateau Scientific Expedition and Research (STEP) program (grant no. 2019QZKK0502), and Wild Plants Sharing and Service Platform of Sichuan Province.

## Conflict of Interest

The authors declare that the research was conducted in the absence of any commercial or financial relationships that could be construed as a potential conflict of interest.

## Publisher’s Note

All claims expressed in this article are solely those of the authors and do not necessarily represent those of their affiliated organizations, or those of the publisher, the editors and the reviewers. Any product that may be evaluated in this article, or claim that may be made by its manufacturer, is not guaranteed or endorsed by the publisher.

## Footnotes

^1^ https://github.com/Fengyaa/Campylotropsis_cp_genome
