# Supplementary material for: Complete Chloroplast Genomes Provide Insights Into Evolution and Phylogeny of Campylotropis (Fabaceae)
Source: Front Plant Sci. 2022 May 18;13:895543. doi: 10.3389/fpls.2022.895543 (PMC9158520; doi:10.3389/fpls.2022.895543)
Supplement: Supplementary file 1 [file Presentation_1.PDF]

Supplementary Material

1 Supplementary data

[see the zip file]

Data S1 Alignment file with raw reads mapping to the assembled *matK* gene in *C. bonii*.

Data S2 Primers and results of sanger sequencing.

2 Supplementary Figures and Tables

2.1 Supplementary Tables

[see the excel file]

Table S1 Information of sampling location and voucher specimen for each individual.

Table S2 GenBank Accession for outgroups used in this study.

Table S3 Statistics of reads mapping to IR junction.

Table S4 Summary statistics of types of SSRs identified in each species.

Table S5 Summary statistics of the distribution of SSRs identified in each species.

Table S6 Summary statistics of long sequence repeat in each species.

Table S7 Raw output information of REPuter for each species.

Table S8 Summary of the Omega and P values in the CODEML analysis for each gene.

2.2 Supplementary Figures

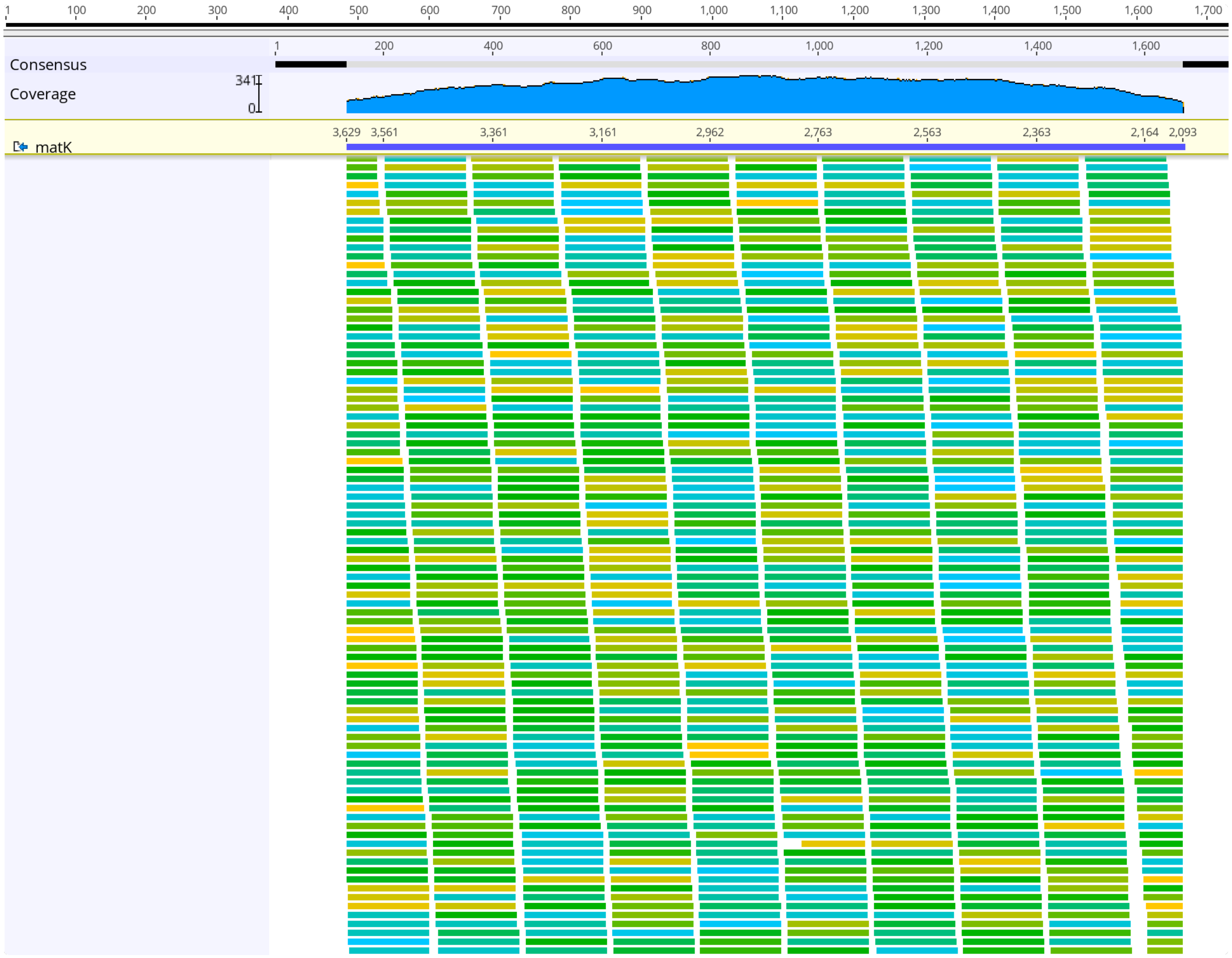

Supplementary Figure 1. Reads mapping for the *matK* pseudogene of *C. bonii*.

Supplementary Figure 2. Comparison of expansion and contraction of IR regions in the 23 cp genomes of *Campylotropisi* and three cp genomes from *Lespedeza maritima*, *Lespedeza cuneata*, and *Kummerowia striata*. [See the .jpg file]

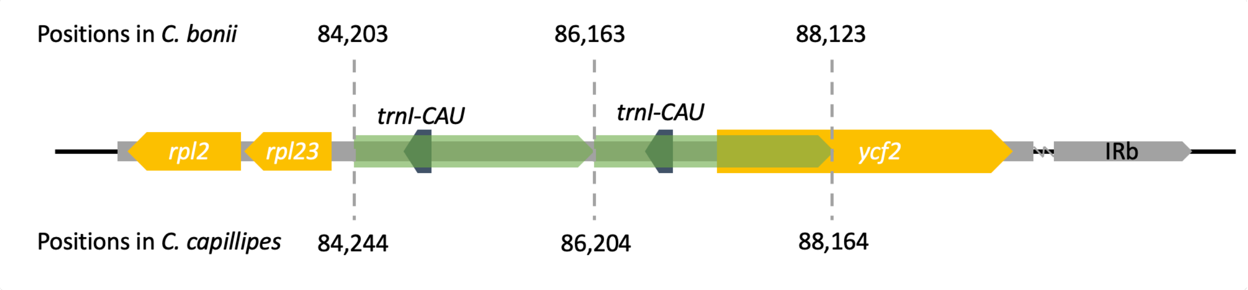

Supplementary Figure 3. Illustration of the long sequence repeat (LSR) occurred in *C. bonii* and *C. capillipes*. The green shadows indicate the LSR region. The dark blue blocks indicate the duplicated *trnI-CAU* gene. The yellow blocks indicate protein-coding genes located surrounding the LSR. The corresponding positions of the LSR in *C. bonii* and *C. capillipes* are indicated at the top and bottom.

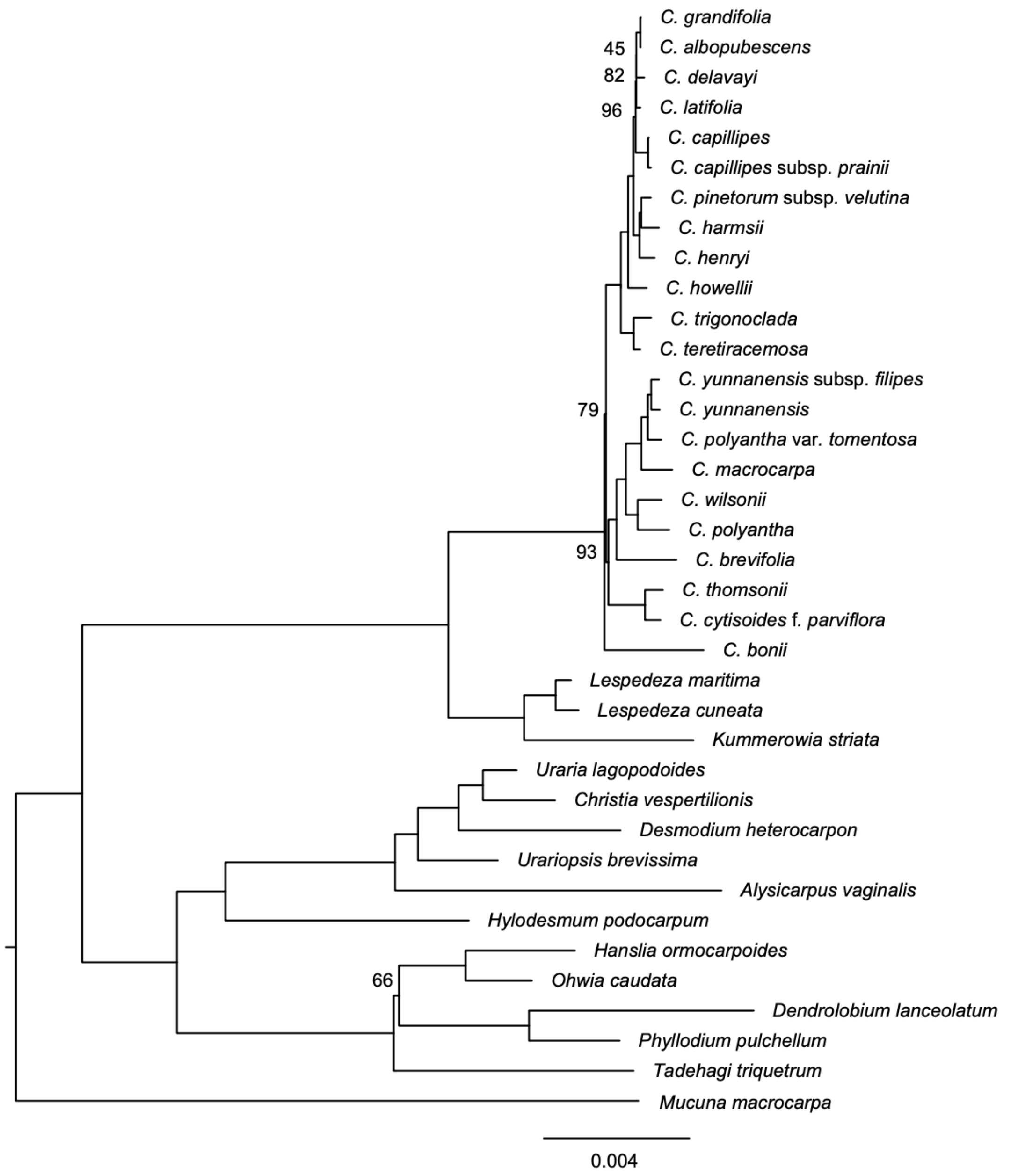

Supplementary Figure 4. Phylogenetic tree obtained using the Maximum Likelihood (ML) method for *Campylotropis* spp. and closely related species based on the concatenated matrix of protein-coding regions. Numbers above branches indicate ML bootstrap supports. The full support values are not indicated.

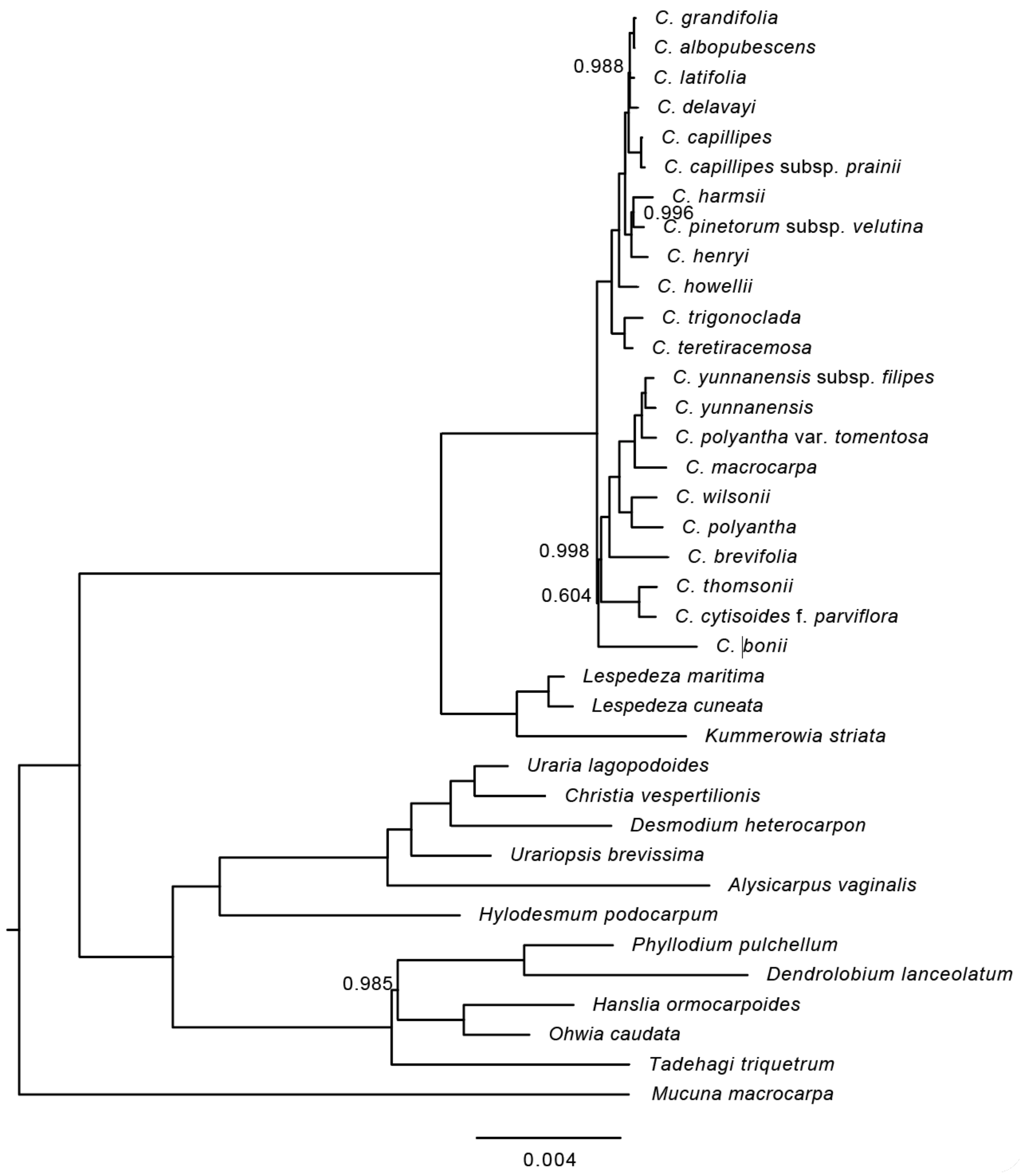

Supplementary Figure 5. Phylogenetic tree obtained using the Bayesian Inference (BI) method for *Campylotropis* spp. and closely related species based on the concatenated matrix of protein-coding regions. Numbers above branches indicate posterior probabilities. The full support values are not indicated.
