# Supplementary figures and images for: Complete Chloroplast Genomes Provide Insights Into Evolution and Phylogeny of Campylotropis (Fabaceae)
Source: Front Plant Sci. 2022 May 18;13:895543. doi: 10.3389/fpls.2022.895543 (PMC9158520; doi:10.3389/fpls.2022.895543)

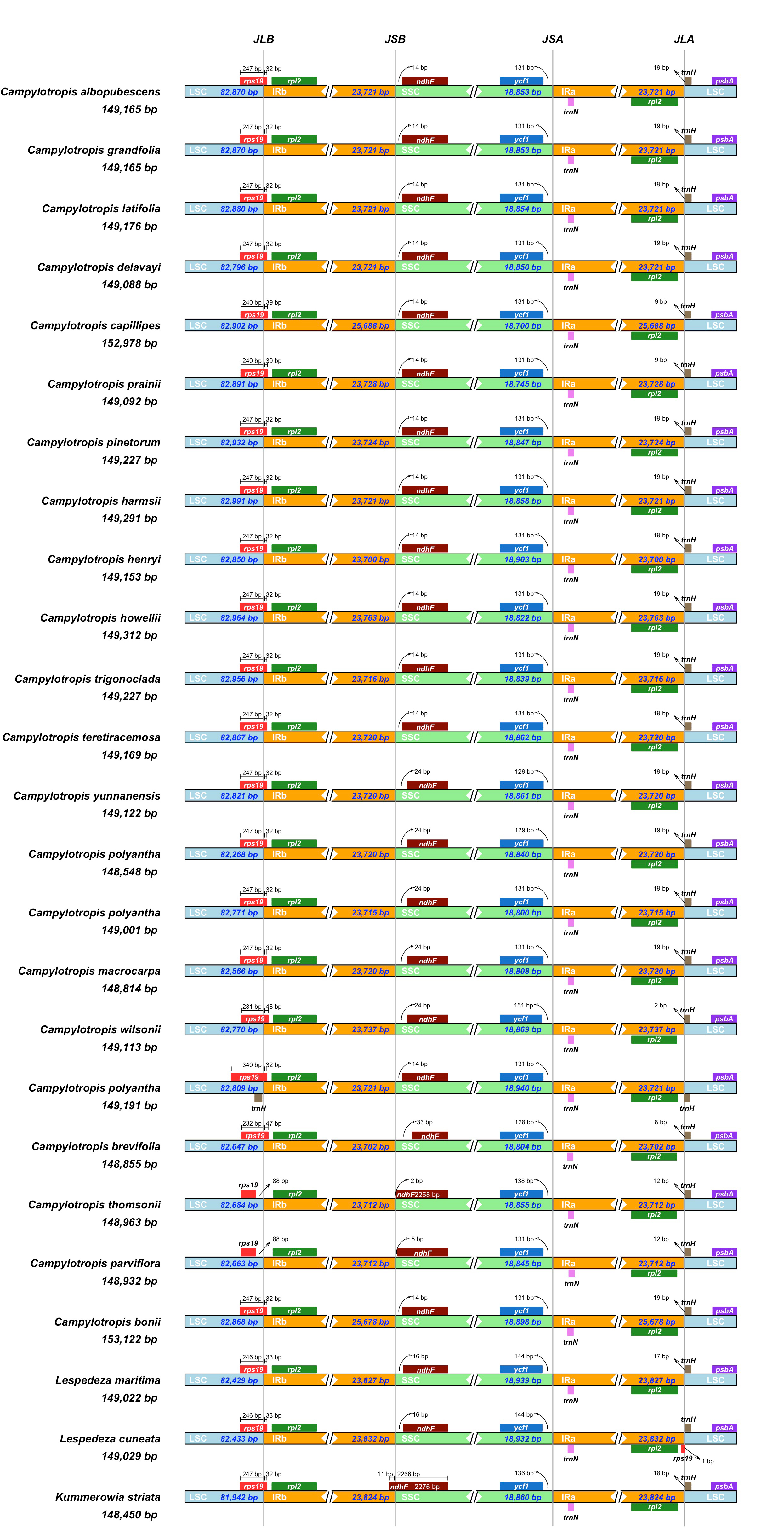

Supplement: Supplementary file 2 [file Image_1.JPEG]
